# Supplementary material for: Interpreting Health Differences between Self-reported Black and White Children in U.S.: Insights from a Methodological Perspective
Source: medRxiv. 2024 Oct 2:2024.10.01.24314712. Preprint. [Version 1] doi: 10.1101/2024.10.01.24314712 (PMC11722458; doi:10.1101/2024.10.01.24314712)
Supplement: Supplement 1 [file NIHPP2024.10.01.24314712v1-supplement-1.pdf]

medRxiv preprint doi: https://doi.org/10.1101/2024.10.01.24314712; this version posted October 2, 2024. The copyright holder for this preprint (which was not certified by peer review) is the author/funder, who has granted medRxiv a license to display the preprint in perpetuity.
This article is a US Government work. It is not subject to copyright under 17 USC 105 and is also made available for use under a CC0 license.

Supplement

Supplementary Figures

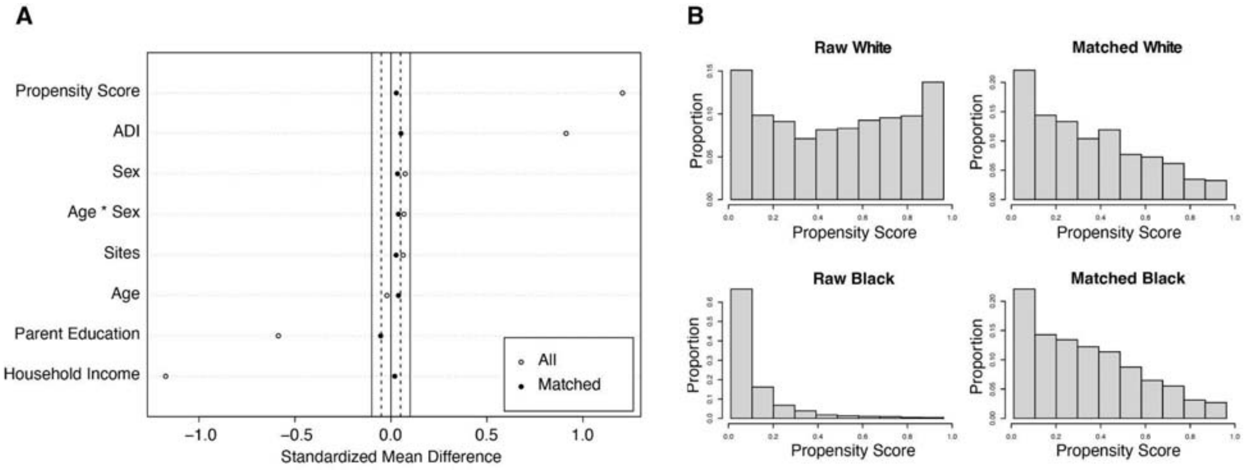

Figure S1. Propensity score matching controls for covariates. (A) The standardized mean differences of covariates between Black and White were reduced below 0.05. (B) The distributions of propensity scores after matching were similar between Black and White. Note: ADI = Area Deprivation Index.
